# Complementary and Alternative Exercises for Management of Osteoarthritis

**DOI:** 10.1155/2011/364319

**Published:** 2011-07-25

**Authors:** Ming-Chien Chyu, Vera von Bergen, Jean-Michel Brismée, Yan Zhang, James K. Yeh, Chwan-Li Shen

**Affiliations:** ^1^Graduate Healthcare Engineering, Whitacre College of Engineering, Texas Tech University, Lubbock, TX 79409, USA; ^2^Department of Mechanical Engineering, Whitacre College of Engineering, Texas Tech University, Lubbock, TX 79409, USA; ^3^Department of Health, Exercise, and Sport Sciences, Texas Tech University, Lubbock, TX 79409, USA; ^4^Department of Pathology, Texas Tech University Health Sciences Center, BB 198, 3601 4th street, Lubbock, TX 79430-9097, USA; ^5^Center for Rehabilitation Research, Texas Tech University Health Sciences Center, Lubbock, TX 79430, USA; ^6^Community and Family Medicine, Texas Tech University Health Sciences Center, Lubbock, TX 79430, USA; ^7^Laura W. Bush Institute for Women's Health, Texas Tech University Health Sciences Center, Lubbock, TX 79430, USA; ^8^Applied Core Laboratory, Winthrop-University Hospital, New York, Mineola, NY 11501, USA

## Abstract

Osteoarthritis (OA) is a chronic condition characterized by degeneration of cartilage and its underlying bone within a joint. With no cure currently available, the goals of treating OA are to alleviate pain, maintain, or improve joint mobility, increase the muscle strength of the joints, and minimize the disabling effects of the disease. Recent research has suggested that complementary and alternative medicine (CAM) exercises may improve OA symptoms. This paper covers CAM mind-body exercises—Tai Chi, qigong, and yoga—for OA management and evaluates their benefits in pain reduction, muscle strength, physical function, stiffness, balance, fear of falling, self-efficacy, quality of life, and psychological outcomes in patients with OA, based on randomized controlled trials published. Findings from the literature suggest that CAM exercises demonstrate considerable promise in the management of OA. Future studies require rigorous randomized controlled trials with larger sample sizes.

## 1. Introduction

Osteoarthritis (OA), the most common joint disorder, is a major cause of disability in the aging population with its prevalence increasing and consequences significantly impacting society [1]. As the population ages worldwide, OA has become a serious health threat to many countries [2]. It is estimated that almost 18% of women and 10% of men 60 years of age and older have symptomatic OA [3]. Eighty percent of those with OA report limitations in movement, while 25% report inability to perform major daily activities of life [3]. The economic costs associated with OA sequelae have increased tremendously in the past decade and are predicted to continue to grow [4]. 

Pain, muscle weakness, and physical dysfunction form a vicious cycle in the knee OA, with muscle weakness being associated with pain and physical dysfunction [5]. Individuals with OA can experience difficulty in walking, balance deficit, and muscle weakness, thus increasing the risk of falling by threefold [6] and consequently, fall-related fractures [7]. Well-established modifiable risk factors of OA include overweight, injury, occupation, structural malalignment, and muscle weakness, while nonmodifiable risk factors include older age, female gender, race, and genetic predisposition [2]. 

There is no cure for OA, as it is very difficult to restore the cartilage [8]. The goals of treatment are to alleviate pain, maintain, or improve joint mobility, increase the muscle strength of the joints, and minimize the disabling effects of the disease [9–13]. Nonpharmacological intervention such as exercise has been emphasized by the medical community [14]. Recent research has suggested that complementary and alternative medicine (CAM) exercises may improve OA symptoms [12, 13]. 

The use of CAM modalities for chronic musculoskeletal disorders, such as OA, is primarily to alleviate associated discomfort and disability, and approximately 50% of patients with chronic musculoskeletal disorders reported using CAM modalities [9]. 

Among different CAM modalities, mind-body therapy is one of the most popular domains in the US [15]. Mind-body exercises can be broken down into subsections of tai chi (TC), qigong, and yoga in the mind-body therapy category covering a wide range of healing practices that share a common intention to improve the mind's capacity to affect bodily function and symptoms [15]. Although significant attention has focused on stretching and strengthening the quadriceps muscles for reducing symptoms of knee OA [16–20], numerous studies have been conducted investigating effects of CAM exercise on OA. The aim of this study is to review the evidence provided by published randomized clinical trials (RCTs) for the effect of TC, qigong, or yoga on various clinical and quality of life outcomes among people with OA. 

## 2. Tai Chi for OA

According to the 2007 National Health Interview Survey (NHIS), an estimated 2.2 million people in the US practice TC for their health [21]. TC is a moderate-intensity mind-body exercise with breath control training to promote vital energy (Qi) and blood circulation [22, 23]. It is a more holistic oriental life cultivation program than a simple physical exercise from a Western point of view. TC features gentle, smooth, graceful, coordinated, and flowing movements of different body parts, emphasizing constant shifting of body weight between two legs with both knees flexed all the time while meditating and breathing deeply [24]. 

People practice TC for various health purposes such as improving physical condition, muscle strength, coordination, flexibility, and balance, decreasing risk for falls, pain, stiffness, and fatigue, and improving sleep, cardiovascular and respiratory function, and overall wellness [25–29]. Tai chi involves slowly stretching the limbs and trunk, requires less physical strength than strenuous exercise, and can be suitable for physically frail older adults to practice in a small space, at any time, individually or in groups, regardless of weather conditions [30]. The National Arthritis Foundation has begun to promote a TC program to improve the quality of life for people with arthritis. A recent systematic review concluded that exercise programs based on TC seems to demonstrated better outcomes in functional aerobic capacity (ability to perform activities of daily living that require sustained aerobic metabolism) than hydrotherapy programs [31]. Another recent systematic review from the same authors concluded that exercise programs based on TC demonstrated better outcomes than mixed exercise programs based primarily on aerobic resistance, strength, and flexibility training in reducing pain in adults with lower limb OA [32]. 

Among all the mind-body exercises in CAM, TC is by far the most studied for the management of OA.  Table 1 summarizes results of all RCT studies, 8 altogether, available in the open literature investigating TC's effects on OA management. All these RCTs are small, with total sample sizes ranging from only 16 participants to 97 participants. RCTs not published in peer-reviewed journals [33] or with too small sample size (<10 people per allocation) [34] are excluded from the present study. 

Various styles of TC were employed for intervention in these studies, including Chen, Yang, Wu/Hao, and Sun styles. Among different TC styles, the “Sun” style is the most studied although it is not the most popular style among TC practitioners worldwide. According to Song et al. [35], Sun-style TC is characterized by slow and continuous movements with follow-up steps and higher stance with bending knees less than other types of TC. This style includes agile steps and exercises that may improve mobility, breathing, and relaxation. The movements do not require deep bending or squatting, which makes it easier and more comfortable to learn [35]. In fact, the characteristics of Sun style TC described above are quite common among all other styles of TC, and most TC can be practiced in high or low stance as the practitioner desires. 

### 2.1. Pain Reduction

Pain is the predominant symptom of OA. Seven RCTs on TC's effects on pain reduction in OA patients have been published [24, 33–40]. Five of the those RCTs reported that TC significantly reduces pain intensity in OA patients, as measured by the Western Ontario and McMaster Universities osteoarthritis index (WOMAC) pain subscale, visual analogue scale (VAS), or pain intensity in SF-36 pain subscale survey, compared to control [40], attention control [26, 39], and usual physical activity [35, 39] (Table 1). However, on the other hand, 2 studies reported that TC provided no benefits in pain reduction with arthritis pain self-efficacy assessment compared to waiting list control [36], and attention control [37]. Both of these 2 studies reporting no benefits by TC in pain reduction involved patients with OA at locations other than the knee, while all those studies focusing only on patients with knee OA demonstrated TC's benefits in pain reduction. This suggests that the slow, gentle, and weight-carrying TC footwork provides pain-relieving effects on OA at the knee, a body part strongly emphasized and required in TC training. Such pain-relieving effect is not significant or even absent at other body parts, particularly those in the upper body, where less weight-carrying activity than knee is involved in TC. TC was also found to result in no difference compared with hydrotherapy in pain reduction in OA patients [36]. However, hydrotherapy requires a major facility (pool), while TC intervention does not need major facility or equipment and can even be implemented at home [24].

The discrepancy of TC's impact on pain reduction in OA patients among different studies cannot be explained by their differences in sample size, intervention duration, frequency and session length, age, or gender. Ethnicity or geography may play a role, as all studies [36, 37] reporting no benefit of TC in OA pain alleviation were conducted outside Asia (Australia and US), while all studies conducted in Korea [35, 39–41] as well as some studies conducted in the US [24, 38] reported positive outcomes. BMI is usually higher outside Asia, and there is probably a higher expectation of positive outcomes/familiarity of TC in Asian communities compared with Caucasian communities. 

### 2.2. Muscle Strength

Stronger muscles and better coordination improve the stability of the knee joint, resulting in less pain [14]. Thus, muscle strengthening is a key component of exercise in the management of knee OA [14]. Studies have established the beneficial effects of exercise in patients with mild-to-moderate OA of the knee, including quadriceps muscle strengthening [14]. 

Only 2 RCTs evaluated TC's effects on muscle strength in OA patients [35, 41], both conducted by the same team. Song et al. [35] reported that compared to telephone-contact control (usual care), TC improved the abdominal muscle strength, and trunk flexion (measured by the number of sit-ups performed in 30 seconds) but made no difference in knee muscle strength and endurance [35] (Table 1). More recently, the same research team reported that extending the TC intervention from 12 weeks to 27 weeks resulted in significantly greater knee extensor endurance, while no difference in knee extensor or flexor strength was observed [41]. These two studies suggest that TC may improve abdominal muscle strength, but not knee muscle strength; it may also improve knee extensor endurance after 6 months of intervention. 

### 2.3. Physical Function

Five RCTs investigating TC's effects on physical function in OA patients have been published [22, 35–38], and 4 of them reported that TC significantly improves physical function in OA patients (Table 1). TC has been shown to improve physical function based on the results of WOMAC-physical function subscale or activity of daily living compared to waiting list control [36], and usual care treatment [35] in subjects with OA-related symptoms. Brismée et al. [24] reported that a 12-week TC program consisting of 6 weeks of group TC followed by 6 weeks of home-based TC improved physical function (WOMAC physical function-subscale) in the elderly with knee OA, relative to an attention control group. Wang et al. [38] reported that compared to the attention control group, 12-week TC intervention improved the physical function, as shown by WOMAC physical function subscale and chair stand time, but TC had no effect on 6-minute walk test performance. 

TC was also found to result in no difference compared with hydrotherapy in improving physical function in OA patients [36], suggesting that TC can provide comparable benefits as hydrotherapy in this respect, but TC intervention requires no equipment. 

### 2.4. Stiffness, Range of Motion, and Flexibility

TC movements involve maintaining some degree of knee flexion while moving in various directions. Such pattern of knee movement is associated with larger peak shear forces and larger peak moments in the lower-extremity joints than normal gait [45]. However, the effects of TC on stiffness, range of motion, and flexibility remain inconclusive. 

Five RCTs on TC's effects on stiffness, range of motion and flexibility in OA patients have been published [24, 38–40] (Table 1). In 4 of these studies, TC significantly reduced joint stiffness compared with control based on WOMAC-stiffness subscale score [35, 38–40], with one of these 4 studies reporting that TC also improved joint motion of knee and knee rising time [40]. On the other hand, one of the 5 RCTs showed that TC made no difference in upper-body flexibility [35], and another study showed that TC made no difference in either stiffness or knee range of motion [24] compared with attention control. Among these 5 RCTs on TC's effects on stiffness in OA patients, 3 were conducted in Korea and 2 in the US. All studies conducted in Korea demonstrated TC's benefit in reducing joint stiffness [35, 39, 40]. One of the 2 US studies showed TC's positive result in stiffness reduction (Wang et al. [38]). The other US study (Brismée et al. [24]) reported no effect, possibly because its TC intervention period (6 weeks of group exercise followed by 6 weeks of home practice) was much shorter compared with that of [38] (12 weeks of group exercise followed by 48 weeks of home practice). 

Regarding joint range of motion, H. Y. Lee and K. J. Lee [40] reported that TC improved knee joint motion and rising time relative to control, whereas Brismée et al. [24] found no difference in knee range of motion for flexion and extension between TC group and attention control in subjects with knee OA. 

### 2.5. Balance and Fear of Falling

One of the most effective prevention strategies for reducing the risk of falling is exercise that focuses on improving muscle strength, balance, and coordination [46, 47]. Women, especially older women with OA, are less likely to participate in any type of physical exercise due to their fear of falling and poor confidence, leading to deconditioning and loss of function [46].

Four RCTs on TC's effects on balance in OA patients have been published [35, 37, 38, 40] (Table 1). In 2 of these studies, TC was shown to significantly improve balance in OA patients compared to usual care [35], and no treatment control [40], measured by one leg standing test. Another study showed that TC tended to improve one-leg balance in OA patients compared to attention control (*P* < 0.1) [37]. However, TC showed no effect on balance relative to attention control in the other study [38] (Wang et al.). Traditional TC involves significant balance training through moves such as the rooster stance featuring one-leg standing, and TC kick featuring standing with one leg while the other leg slowly kicks up and lowers down in a period of about 5 seconds or longer. TC footwork requiring constantly moving the body in different directions in a slow and controlled fashion with one supporting leg also provides remarkable balance training. However, if these balance training moves and elements are not included in the exercise intervention, the beneficial effect of TC on balance may not show. 

Two RCTs on TC's effects on falling in OA patients have been published [40, 41] (Table 1). These studies reported that TC decreased fear of falling in OA patients compared to self-help education control [41] and no treatment control [40]. On the other hand, the study [40] also showed no impact due to TC on disability and fall efficacy. 

### 2.6. Self-Efficacy

Two RCTs on TC's effects on self-efficacy in OA patients have been published [37, 38] (Table 1). These studies reported that TC improved self-efficacy for arthritis symptoms and total arthritis self-efficacy relative to attention control. On the other hand, Song et al. [39] reported no difference in perceived self-efficacy including perceived benefits/barriers and emotional salience, assessed by motivation scale for health behaviors, between TC and usual care. 

### 2.7. Quality of Life

The effect of TC on QOL in OA patients has been evaluated in 3 RCTs [36–38] (Table 1). Among these 3 studies, TC benefited QOL in OA patients in 2 of them, while the other showed no such effect of TC. Hartman et al. [37] reported that TC favored the satisfaction with general health including improved walking speed, bending ability, arm function, self-care activities, and household tasks with arthritis impact measurement scale (AIMS) survey [47]. Wang et al. [38] showed that TC improved health-related QOL measured by SF-36 [49] survey in patients with knee OA. On the other hand, Fransen et al. [36] reported no difference in changes in the physical and mental component summary scores of the SF-12 [50] among TC, hydrotherapy program, and waiting list control. 

### 2.8. Psychological Outcomes

Three RCTs investigating TC's effects on psychological outcomes in OA patients have been published [36, 38, 39], with 2 of them reported positive results (Table 1). Wang et al. reported that TC improved depression symptoms, assessed by Center for Epidemiologic Studies depression scale, compared to attention control [38]. TC made OA patients perceive more health benefits and perform better health behaviors, especially with diet and stress management, assessed by health behavior scale [39]. On the other hand, TC resulted in no difference in depression, anxiety, and stress, measured by DASS 21, in OA patients [36]. 

## 3. Qigong Exercise for OA Management

Qigong (Chi Gong or Chi Kung) is an art of moving qi/chi (vital life energy) through the body, releasing energy blocks, and eliminating causes of illness and imbalance. Hundreds of forms of qigong exercises designed for specific or general health enhancement purposes have been created and practiced [51]. According to traditional Chinese medicine, good health is the result of free-flowing, well-balanced qi, while sickness or pain, such as arthritis, is the result of a blockage of the qi flow or unbalanced qi in the body [42, 51, 52]. 

Qigong can be divided into internal and external types. Internal qigong refers to individual practices and exercises to achieve optimal dynamic mind-body integration through improved qi circulation such as TC qigong, Baduanjin qigong, and meditation. External qigong involves interaction between a patient and a qigong healer who uses hand movement, acupressure on specific points, focused attention, and/or projection or emission of qi toward the patient's body to improve the flow of qi by breaking qi blockages or removing sick qi, to restore balance of the system, to relieve pain, and/or to cure disease [53–55].

External qigong therapy (EQT) and internal qigong practice may palliate symptoms of arthritis by relaxing diseased tissues and enhancing blood flow to the area [42, 56–58]. Increased blood flow leads to more efficient delivery of oxygen, nutrients, pain-killing substances and drugs, as well as more efficient removal of mediators of pain and metabolic waste products that contribute to pain [57, 58]. There is considerable variability in the EQTs of different schools and practitioners. Some studies in China reported improvement of severe arthritis symptoms by EQTs [42]. 

Three RCT studies evaluating the impact of qigong on management of OA have been reported (Table 2). Chen et al. [42] reported that OA subjects in the EQT group, after receiving 5 to 6 sessions of EQT treatment in 3 weeks by two different healers, demonstrated a greater pain reduction and improvement in functionality (as assessed by WOMAC) than those in the placebo-sham group. It was further found that the beneficial effects of EQT on pain reduction and functionality improvement were sustained 3 months after the intervention, but only in subjects treated by one of the 2 healers (healer 2). Subjects treated by this healer also tended to walk faster than the placebo-sham group, but similar result was not observed in those subjects treated by the other healer (healer 1). There was no difference in range of motion, anxiety or depression scale between the EQT group and the sham-control group, regardless of healers. Subjects treated by healer 2 tended to demonstrate lower negative mood level but not in those treated by healer 1. Apparently, the results of the two qigong healers were not consistent, and outcomes of healer 2 seemed to be more beneficial than healer 1, making the results difficult to interpret. This suggests that the effectiveness of EQT may be provider dependent and underscores the difficulty of EQT research as different qigong healer (master) may possess different level of healing qi power that may result in different outcomes in patients. Even the qi power of the same healer may vary depending on his/her own physical condition at the time of treatment. The basic unresolved issue is quantification of the power of qi that is delivered by the healer and actually received by the patient through physical measurements. Before this issue is resolved, EQT clinical studies will continue to be limited by unquantified intervention. 

In internal qigong, *Baduanjin qigong *(translated as the “eight section of brocades”, as it contains 8 fine, delicate, and smooth exercise movements) can be learned easily and is less physically and cognitively demanding than TC [43]. *Baduanjin* was defined as a low-level aerobic exercise that can improve the limbs' range of motion, strength, and general health [43], while it also contains much stretching and controlled breathing aimed to improve circulation of qi. In a pilot feasibility study by An et al. [43], subjects with OA reported reduced knee pain and stiffness, and improved physical function in the *Baduanjin* group compared to the control group (Table 2). In addition, subjects in the *Baduanjin* group demonstrated statistically significant improvement in aerobic capacity (assessed by 6-minute walking test) as well as quadriceps strength (assessed by isokinetic strength of the knee extensors) [43]. However, there was no difference in SF-36 between the *Baduanjin* group and the control group (Table 2). 

TC qigong, another form of internal qigong, is a combination of TC movement with qigong training including incorporating intention into movement and enhancing awareness of breathing [44]. Basically, TC qigong can be considered as TC focusing on qi and without martial applications. The TC qigong implemented in [44] consisted of a sequence of 18 movements, performed six times in a fluid and continuous manner, combined with deep abdominal breathing. It was claimed that TC qigong is an easy and safe qigong for patients, because it is simpler and more repetitive than TC, and it is especially suitable for patients with joint problems, as the motion does not impose undue pressure on the pivot joint, such as the waist, knee, and ankles during training [44]. TC qigong improved physical function (measured by 6-min walking test) and quality of life (measured by SF-36) compared to the waiting list control [44]. 

## 4. Yoga Exercise for OA Management

Yoga is a CAM mind-body practice, with origins in ancient Indian philosophy, that combines physical postures, breathing techniques, and meditation or relaxation. A 2008 survey of 5,050 adults indicated that 6.9% of U.S. adults, or 15.8 million people, practice yoga [59]. Among numerous styles of yoga, Hatha yoga is the most commonly practiced in the United States and Europe. Hatha yoga was originally designed as a preparatory practice for physical purification before meditation and has been adopted in the Western civilization as a therapy to increase flexibility, strength, and stamina, while also promoting self-awareness, emotional stability, and peace of mind. Yoga implements controlled postures, breathing and meditation to achieve these goals [60, 61]. It usually involves holding the body in a sequence of postures for a certain period of time, breathing exercises, and meditation. 

The practice of Hatha yoga has been shown to reduce pain, relieve tension, reduce risks of injury, improve posture, improve communication, increase energy and attention span, and enhance feelings of overall wellness and well-being [62]. The use of Iyengar approach to Hatha yoga emphasizes strength, flexibility, and relaxation, with particular attention to alignment of body structures (e.g., the relationship of the distal to the proximal extremities and the extremities to the spine and torso) [48, 63, 64]. Raman suggested that lyengar method of Hatha yoga can be used as a supplement to other measures to treat musculoskeletal problems [65]. Yoga is one of several practices that have the potential to be beneficial to OA [66, 67]. 

There is only one RCT study published evaluating the impact of yoga on management of OA (Table 3). In their RCT of patients with hand OA, Garfinkel et al. showed that yoga exercise significantly decreased finger pain during activity, improved finger range of motion, but showed no difference in grip strength or joint circumference compared to the waiting list control [66]. 

## 5. Conclusion and Future Research

Findings based on only a small number of RCTs available in the literature suggest that CAM exercises—Tai Chi, qigong, and yoga—demonstrate considerable promise in the management of OA symptoms. In particular, it has been shown that TC may reduce arthritic pain, enhance physical functioning and flexibility, and improve self-efficacy and quality of life in OA patients. However, most existing studies suffer methodological limitations (e.g., small sample size, nonrandomized trial, inconsistent intervention, short intervention duration, inconsistent instruments for outcomes assessment, and short follow-up periods) that hinder interpretation of findings and preclude firm conclusions. Further, there may be reasons for false positive results, such as participants unblinded to allocation, Hawthorne effect multiple outcomes measured. Future studies require rigorous and high-quality randomized controlled trials with larger sample sizes along with standardized and quantified intervention and quantitative assessments for pain reduction, muscle strength and endurance, physical performance, joint motion and stiffness, flexibility, and OA-related biofluid biomarkers that have been shown to be reliable and responsive to change due to exercises. 

## Figures and Tables

**Table 1:** Effects of Tai Chi on management of osteoarthritis reported in randomized controlled trials.

First author (year) [ref.]	Subjects	Exercise intervention	Control intervention	Results
Brismée (2007) [24]	Older persons (≥50 yr) with OA at knee in US, *N* = 41 with 18% attrition in TC and 32% attrition in attention control.	24-form Yang style TC (40 min/session × 3 sessions/week × 6 weeks followed by home-based TC practice at 3 sessions/week × 6 weeks), *N* = 22 (70.8 ± 9.8 yr).	Attention control program (40 min health lecture × 3 lectures/week × 6 weeks followed by no activity for 6 weeks), *N* = 19 (68.8 ± 8.9 yr).	Compared to the attention control group, TC group (i) reduced pain (VAS, WOMAC), (ii) improved physical function (WOMAC), (iii) showed no difference in stiffness (WOMAC), (iv) showed no difference in knee range of motion for flexion and extension assessed by goniometry.

Song (2003) [35]	Older women (≥55 yr) with OA at multiple sites in South Korea, *N* = 72 with 43% attrition in TC, 39% attrition in control.	12-form Sun style TC modified for arthritis (60 min/session × 3 sessions/week × 2 weeks, then 60 min/session × 1 session/week × 10 weeks), *N* = 22 (64.8 ± 6.0 yr).	Telephone-contact control (usual care), *N* = 21 (62.5 ± 5.6 yr).	Compared to the control group, TC group (i) reduced pain (K-WOMAC), (ii) showed no difference in knee muscle strength and endurance, (iii) improved trunk flexion and abdominal muscle strength (increased frequency of situps), (iv) perceived fewer difficulties in physical functioning of daily living (ADL), (v) decreased joint stiffness (K-WOMAC), (vi) showed no difference in upper-body flexibility, (vii) improved balance by standing longer on one foot.

Fransen (2007) [36]	Older persons (ages 59–85 years) with OA at hip or knee in Australia, *N* = 152 with 7% attrition in TC, 5% attrition in hydrotherapy, no dropout in control.	24-form Sun style TC modified for arthritis (60 min/session × 2 sessions/week × 12 weeks), *N* = 56 (70.8 ± 6.3 yr).	Waiting list control, *N* = 41 (69.6 ± 6.1 yr).	Compared to the waiting list control group, TC group (i) showed no difference in pain reduction (WOMAC), (ii) improved physical function (WOMAC), (iii) showed no difference in quality of life (SF-12), (iv) showed no difference in depression, anxiety, and stress (DASS 21).

Hartman (2000) [37]	Older person with OA at multiple sites (hip, knee, ankles, foot) in US, *N* = 35 with 6% attrition in TC.	9-form Yang style TC (60 min/session × 2 sessions/week × 12 weeks), *N* = 17 (68.6 ± 7.9 yr).	Attention control program (usual physical activity, routine care, total 3 times group meeting, and telephone discussion every 2 weeks), *N* = 16 (67.5 ± 6.1 yr).	Compared to the control group, TC group (i) showed no difference in pain reduction (arthritis pain self-efficacy), (ii) reduced level of tension (AIMS II), (iii) improved self-efficacy for arthritis symptom (Arthritis Self-Efficacy Scale) and total arthritis self-efficacy, (iv) improved satisfaction with general health including improved walking speed, bending ability, arm function, self-care activities, and household tasks (AIMS II).

Wang (2009) [38]	Older persons (≥55 yr) with OA at knee in US, *N* = 40 with no dropout.	10-form Yang style TC (60 min/session × 2 sessions/week plus 20 min/day home-based TC for 12 weeks, followed by home-based TC practice for 48 weeks), *N* = 20 (63 ± 8.1 yr).	Attention control program (60 min/session of health lecture plus stretching exercise × 2 sessions/week × 12 weeks), *N* = 20 (68 ± 7.0 yr).	Compared to the attention control group, TC group (i) reduced pain (WOMAC, VAS), (ii) improved physical function (WOMAC, chair stand time), (iii) showed no difference in performance for 6-minue walking test, (iv) reduced stiffness (WOMAC), (v) showed no difference in balance score, (vi) improved self-efficacy, (vii) improved health-related quality of life (SF-36), (viii) improved depression scale (Center for Epidemiologic Studies depression scale).

Song (2007) [39]	Older women (≥55 yr) with OA at multiple sites in South Korea, *N* = 72 with 43% attrition in TC, 39% attrition in control.	12-form Sun style modified TC for arthritis (60 min/session × 3 sessions/week × 2 weeks, then 60 min/session × 1 session/week × 10 weeks), *N* = 22 (64.8 ± 6.0 yr).	Telephone-contact control (usual care), *N* = 21 (62.5 ± 5.6 yr).	Compared to the control group, TC group (i) reduced pain (K-WOMAC), (ii) decreased joint stiffness (K-WOMAC), (iii) showed no difference in perceived self-efficacy including perceived benefits/barriers and emotional salience (motivation scale for health Behaviors), (iv) perceived more health benefits, (v) performed better health behaviors, especially for diet behavior and stress management (health behavior scale).

H. Y. Lee (2008) [40]	Older person with OA at knee in Korea, *N* = 46 (75.4 ± 6.2 yr).	24-form Sun style TC (60 min/session × 2 sessions/week × 12 weeks), *N* = 22.	No treatment, *N* = 24.	Compared to the control group, TC group (i) reduced pain (WOAMC), (ii) reduced stiffness (WOAMC), (iii) improved joint motion of knee and knee rising time, (iv) improved balance (single leg test), (v) decreased fears of falling, (vi) showed no difference in disability and falls efficacy.

Song (2010) [41]	Older women (≥55 yr) with OA at multiple sites in South Korea, *N* = 82 with 24% attrition in TC, 15% attrition in control.	31-form Wu style TC modified for arthritis (60 min/session × 2 sessions/week × 3 weeks, then 60 min/session × 1 session/week × 6 months), *N* = 30 (63.0 ± 7.2 yr).	Self-help education program (2 hours/session × 1 session/month × 6 months), *N* = 35 (61.2 ± 7.9 yr).	Compared to the control group, TC group (i) improved knee extensor endurance, (ii) showed no difference in knee extensor and flexor strength, (iii) reduced fear of falling during daily activities.

ADL: activities of daily living; AIMS: Arthritis Impact Measurement Scale; TC: Tai Chi; WOMAC: Western Ontario and McMaster Universities Osteoarthritis Index; VAS: visual analogue scale.

**Table 2:** Effects of Qigong exercise on management of osteoarthritis reported in randomized controlled trials.

First author (year) [ref.]	Subjects	Exercise intervention	Control intervention	Results
Chen (2008) [42]	Older persons (≥50 yr) with knee OA in US, *N* = 112 with 5% attrition in qigong, 5% attrition in placebo-sham control.	External qigong therapy by two healers (5-6 sessions in 3 weeks), *N* = 45 (63.9 ± 9.7 yr for healer 1) and *N* = 12 (58.8 ± 7.0 yr for healer 2).	Placebo-sham by two healers (mimicked external qigong therapy, 5-6 sessions in 3 weeks), *N* = 52 (62.9 ± 9.2 yr).	Compared to the placebo-sham group (1 and 3 months postintervention followup), external qigong group (i) decreased pain (WOMAC) in healer 1 and healer 2, (ii) improved functionality (WOMAC) and total WOMAC in healer 2, (iii) showed no difference in range of motion, (iv) showed no difference in psychological outcomes including anxiety level (Spielberger state trait anxiety scale) and depression scales (Center for Epidemiologic Studies depression scale).

An (2008) [43]	Older persons with knee OA in China, *N* = 28 with 21% attrition in Baduanjin and 28% attrition in control.	Baduanjin (30 min/session × 5 sessions/week × 8 weeks), *N* = 14 (65.4 ± 8.2 yr).	No treatment, *N* = 14 (64.6 ± 6.7 yr).	Compared to the control group, Baduanjin group (i) reduced pain and stiffness (WOMAC), (ii) improved physical function (WOMAC), (iii) improved aerobic capacity (6-minute walk test) and Peak Torque of quadriceps (isokinetic strength of the knee extensors), (iv) showed no difference in general health, social function, and mental health subscales (SF-36).

Lee (2009) [44]	Older person with knee OA in Korea, *N* = 44 with 3% attrition in TC qiqong and 13% in control.	TC qigong (60 min/session × 2 sessions/week × 8 weeks), *N* = 28 (70.2 ± 4.8 yr).	Waiting list control, *N* = 13 (66.9 ± 6.0 yr).	Compared to the control group, TC qigong group (i) improved physical function by 6-min walking test, (ii) improved quality of life (SF-36).

TC: Tai Chi; WOMAC: Western Ontario and McMaster Universities Osteoarthritis Index.

**Table 3:** Effects of yoga exercise on management of osteoarthritis reported in a randomized controlled trial.

First author (year) [ref.]	Subjects	Exercise intervention	Control intervention	Results
Garfinkel (1994) [48]	Older persons (≥50 yr) with OA at hands (fingers) in US, *N* = 25 with 4 attritions in total	Yoga + relaxation + education (60 min/session × 1 session/week × 8 weeks), *N* = 17	Waiting list control, *N* = 8	Compared to the waiting list control, yoga group (i) decreased pain, (ii) improved range of motion, (iii) showed no difference in grip strength or joint circumference.
